# Ciprofloxacin adsorption to magnetite-pine bark biosorbents as affected by preconditioning with distinct microbiomes

**DOI:** 10.1007/s11356-026-37778-w

**Published:** 2026-04-30

**Authors:** Mahdiyeh Mohammadzadeh, Simon Bo Lassen, Mia Kristine Staal Jensen, Bjarne Westergaard Strobel, Kristian Koefoed Brandt, Tiina Leiviskä

**Affiliations:** 1 Chemical Process Engineering, University of Oulu, P.O. Box 4300, FIN-90014 Oulu, Finland; 2 Department of Plant and Environmental Sciences, University of Copenhagen, Thorvaldsensvej 40, DK-1871 Frederiksberg C Copenhagen, Denmark; 3 Sino-Danish Center for Education and Research (SDC), Beijing , 100049 China

**Keywords:** Adsorption, Magnetite-pine bark, Antibiotics, Wastewater treatment, Bacterial preconditioning, 16S rRNA gene amplicon sequencing

## Abstract

Biosorbents from agricultural and forestry waste are low-cost, eco-friendly, and biodegradable, offering abundant availability, reusability, and a low risk of secondary pollution. However, factors such as microbial colonisation can change their surface properties and affect adsorption efficiency. This study investigated how biosorbent loading with microorganisms affected the adsorption efficiency of pharmaceuticals. The adsorption efficiency of ciprofloxacin (CIP) on magnetite-pine bark (MPB) biosorbent was evaluated after preconditioning with either a population of specific bacterial cells (*Aeromonas rivipollensis* strain isolated from municipal wastewater) or hospital wastewater effluent harbouring a complex microbial community. The removal efficiency trends after exposure to pure culture bacteria at three timepoints of 3 h, 24 h, and 48 h showed more decline (5.1–56.6%) in performance compared to those (3.7–42.9%) observed after exposure to wastewater effluent at three timepoints of 3 h, 24 h, and 1 week. In most cases, 24-h bacterial or wastewater colonisation reduced adsorption efficiency to (18.2–56.6%) and (3.7–21.3%) in the order at contact times 10–360 min. According to 16S rRNA gene amplicon sequencing, *Pseudomonas* was the most abundant genus at all three distinct colonisation times (24 h, 48 h, and 1 week). Microbial diversity on the MPB increased progressively over time after being exposed to hospital wastewater over 24 h to 1 week colonisation times and bacteria from the hospital wastewater colonised the biosorbent. Collectively, these findings suggest that complex microbial communities in wastewater contribute both positively and negatively to adsorption performance.

**Supplementary Information:**

The online version contains supplementary material available at 10.1007/s11356-026-37778-w.

## Introduction

Antibiotics are widely used to treat infections and prevent diseases in humans and animals (Van Hoek et al. 2011). The release of antibiotics unmetabolized into the environment contributes to the development of antibiotic-resistant bacteria (ARB) and antibiotic resistance genes (ARGs) (Ben et al. 2019). Excessive use of antibiotics leads to antimicrobial resistance (AMR), allergic reactions, toxicity, and neurological or psychiatric issues, particularly with some antibiotic categories such as fluoroquinolones and sulfonamides (MacGowan and Macnaughton 2017). Fluoroquinolones (FQs) are broad-spectrum antibiotics, and CIP shown in Table 1 is a widely used FQ (Bhatt and Chatterjee 2022). CIP has been detected in surface water at concentrations ranging from 2.45 × 10^−4^ mg/L (Rodrigues-Silva et al. 2014) to 31 mg/L (Larsson et al. 2007), and in hospital effluent, levels have been reported between 7.0 × 10^−4^ mg/L and 0.1245 mg/L (Nunes et al. 2018). CIP is one of the pharmaceutically active compounds commonly found as a major contaminant in effluents from the pharmaceutical industry (Gupta and Garg 2018). Conventional wastewater treatment processes can only partially eliminate CIP, primarily through its adsorption onto sewage sludge (Zhu et al. 2015). Residual CIP in water, even at low levels, can lead to ARGs, posing a serious threat to aquatic life and human health (Zhang et al. 2022). Therefore, due to high concentrations of CIP in various wastewater sources, along with its resistance to degradation and potential ecotoxic effects, the effective removal of CIP is necessary (Yan et al. 2019).

**Table 1:** Chemical properties of CIP

Antibiotic	3D chemical structure	Chemical formula	Molecular weight (g/mol)	pKa
Ciprofloxacin (CIP)		C_17_H_18_FN_3_O_3_	331.34	5.9 and 8.9

Various water treatment methods, such as membrane separation (Qalyoubi et al. 2022), advanced oxidation (Mondal et al. 2018), photocatalytic degradation (Feng et al. 2024), and adsorption (Pham et al. 2024), have been utilised to remove CIP. Among these, adsorption has the advantages of ease of use, low operational cost, small environmental impact, and high efficiency (Nassar et al. 2019). Biosorbents, derived from agricultural and forestry waste, are cost-effective, eco-friendly, and biodegradable (Cui et al. 2022), with advantages such as abundant availability, reusability, and minimal risk of generating secondary pollutants in aquatic systems (Huang et al. 2020). However, the performance of adsorbents can be influenced by various factors, including microbial colonisation, which may alter surface properties and adsorption performance. It was reported earlier that the presence of microorganisms enhanced nitroimidazole adsorption on activated carbon (Rivera-Utrilla et al. 2009), most likely by forming biofilms that change texture and surface charge of carbon (Rivera-Utrilla et al. 2003) and prolong carbon bed life (Wilcox et al. 1983). However, it was reported that microorganisms reduce external surface area due to pore blocking and decrease pH_pzc_ (Rivera-Utrilla et al. 2003; Rivera‐Utrilla et al. 2001), while increasing hydrophobicity, which favours adsorption efficiency (Rivera-Utrilla et al. 2009). Recent research (Moral-Rodriguez et al. 2020) showed that adhesion of *E. coli* bacteria to the surface of carbon xerogel (XCs-500) increased the adsorption capacity of diclofenac by approximately 1.3 times. However, studies examining how microbial exposure of adsorbents and different colonisation timepoints influence the adsorption performance are limited, and there is a need for further investigation of the effect of bacteria on the adsorption performance of pharmaceuticals. This study enhances the understanding of microbial-physicochemical interactions in biosorption, offering valuable insights for the long-term design and optimisation of wastewater treatment systems.

The primary aim of the present research was to investigate the impact of bacterial exposure of magnetite-pine bark (MPB) biosorbent on CIP adsorption performance using two microbial preconditioning approaches: (1) the *Aeromonas rivipollensis* strain isolated from municipal wastewater under controlled microbial conditions and (2) hospital wastewater effluent treated with a membrane bioreactor (MBR), representing a complex microbial community. Additionally, the composition of the bacterial community colonising the biosorbent was investigated using microbiome analysis. MPB was selected as a biosorbent because it showed effective performance for pharmaceuticals adsorption in the previous studies both in batch (Mohammadzadeh and Leiviskä, 2023) and pilot-scale column modes (Mohammadzadeh et al. 2025). *Aeromonas* species are frequently isolated from surface waters, a prevalence linked to elevated levels of total organic carbon and assimilable organic carbon. As a result, they may serve as effective indicators of water pollution and overall water quality (Figueira et al. 2011). This study investigates adsorption efficiency under different microbial exposure conditions and at three different timepoints of 3 h, 24 h, and 48 h for pure culture bacteria exposure and 3 h, 24 h, and 1 week for wastewater exposure and also identifies the type of microbes colonising the biosorbent after exposure to real wastewater effluent. The effects of preconditioning time, contact time, and the initial concentration of CIP on adsorption efficiency were examined. The adsorption kinetics and isotherms were modelled using the experimental data.

## Materials and methods

### Raw materials and reagents

Pine bark (from the company RoihuPuu) was rinsed with de-ionised water, and then, it was dried (80 °C, 24 h), crushed, and sieved to achieve a fraction of 250–500 µm. The iron salts (FeSO_4_.7H_2_O and FeCl_3_.6H_2_O) were obtained from J.T.Baker and Acros Organics in that order. The NaOH pellets from VWR Chemicals were used to prepare a 1 M NaOH solution. Milli-Q water from Merck Millipore was used to prepare reagents. CIP (> 98%, Sigma-Aldrich) was used to prepare the synthetic solutions by dissolving the antibiotic in Milli-Q water. Hospital sewage effluent was obtained after the membrane bioreactor (MBR) at the Herlev Hospital wastewater treatment plant (Herlev, Denmark). The wastewater effluent was kept in a cold room prior to use within a week. This hospital wastewater effluent contains residual pharmaceuticals and other organic compounds (the exact composition was not analysed in this study). After MBR, the effluent is still treated with activated carbon, ozone, and UV at this hospital wastewater treatment plant.

### Preparation of magnetite-pine bark (MPB) biosorbent

Magnetite-pine bark (MPB) was prepared as described previously (Mohammadzadeh and Leiviskä, 2023). In brief, pine bark, obtained from RoihuPuu Oy in Finland, was dried and sieved to a fraction of 250–500 µm. Then, the dried pine bark was soaked in Milli-Q water to form a suspension. A solution of iron salts (Fe^3+^:Fe^2+^ = 2:1) and 1 M NaOH were added dropwise to the pine bark mixture. The pH of the mixture was adjusted to 9–10. Then, the mixture was heated (70 °C, 3 h) and afterwards washed with de-ionised water. MPB was obtained after it was dried (60 °C, 24 h). The procedure was repeated six times to obtain a sufficient amount of biosorbent.

### Preconditioning MPB biosorbent with bacteria or hospital wastewater effluent

Prior to batch adsorption experiments (Sect. “Batch adsorption experiments over MPB with CIP synthetic solutions”), MPB biosorbent was preconditioned either with a defined suspension of bacterial cells (first experiment) or with a complex microbial community derived from Herlev Hospital MBR effluent (second experiment).

#### Preconditioning with pure culture bacteria

In the first experimental setup, the MPB biosorbent was preconditioned with a defined suspension of bacterial cells belonging to the species *Aeromonas rivipollensis* prior to adsorption tests. The used bacterial strain was previously isolated from the effluent of the Lynetten municipal wastewater treatment plant in Copenhagen, Denmark (Xu et al. 2025). The strain was revived from a cryostock on Ampicillin Dextrin Agar (ADA) plates and incubated overnight at 28 °C. A single bacterial colony was taken from the plate using a sterile inoculation loop, transferred to Reasoner’s R2 (R2A) liquid medium (35 mL), and incubated overnight at 28 °C. The stationary phase cells were harvested by centrifugation (5000 × g) and washed with 1/10 strength R2A medium. Subsequently, the culture was re-centrifuged and diluted in fresh 1/10 R2A medium to reach an optimal density (OD_600nm_) of 0.1 using a spectrophotometer (Shimadzu). Finally, MPB (1 g/L) was added to the bacterial suspension. The biosorbent was preconditioned (shaking, 250 rpm, 21 °C) with the bacterial solution for different timepoints: 0 h (control: represents samples without preconditioning with bacteria or wastewater effluent), 3 h, 24 h, and 48 h. After the biosorbent preconditioning period, the bacterial suspension was carefully removed by pipetting, leaving behind only the preconditioned biosorbent in the container to be used for the batch adsorption experiments described in Sect. “Batch adsorption experiments over MPB with CIP synthetic solutions”.

The number of viable cells was determined by counting colony-forming units (CFUs), and 100 µL samples were plated onto R2A agar plates (VWR) for colony-forming units (CFUs) after incubation for 1 h and 3 h. The CFUs were counted after 24 h assuming more than 200 colonies as the countable number of colonies.

#### Preconditioning with hospital wastewater MBR effluent

In the second experimental setup, MPB biosorbent was preconditioned with a complex assemblage of microbial cells derived from Herlev Hospital MBR effluent before adsorption. For this purpose, MPB (1 g/L) was added directly to the MBR effluent and preconditioned (250 rpm, 21 °C) for 0 h (control), 3 h, 24 h, and 1 week (168 h). After the biosorbent preconditioning period, the bacterial suspension was carefully removed by pipetting, leaving behind only the preconditioned biosorbent in the container to be used for the batch adsorption experiments described in Sect. “Batch adsorption experiments over MPB with CIP synthetic solutions”.

### Batch adsorption experiments over MPB with CIP synthetic solutions

Batch adsorption experiments were performed with synthetic solutions of CIP and magnetite-pine bark at a dosage of 1 g/L. The experiments were conducted in triplicate (*n* = 3) at room temperature (~ 21 °C). In each trial, 10 mL of a CIP solution (initial antibiotic concentration = 1.6, 5.9, and 10.3 mg/L, pH unadjusted) was added to 0.01 g of bacteria/wastewater-preconditioned biosorbent undried in the same tube used for preconditioning to avoid material loss. The mixture was shaken for 24 h at 250 rpm. The supernatant was collected with a pipette and then frozen for analysis. The pH of the antibiotic solution was measured using a pH meter (edge multi pH meter, Hanna Instruments). The CIP concentrations were analysed using liquid chromatography-mass spectrometry (LC-MS) from Waters UPLC Acquite Iclass MS xevo TQD with a column from Waters ACQUITY UPLC CSH C18 1.7 µm 2.1 × 50 mm. The mobile phases were composed of water with 0.1% (V/V) formic acid and acetonitrile with 0.1% (V/V) formic acid. The injection volume was set at 5 µL. To investigate the effect of contact time, the batch shaking duration was varied between 10 min, 1 h, 3 h, 6 h, and 24 h (initial antibiotic concentration = 1.6, 5.9, and 10.3 mg/L, pH unadjusted). To examine the effect of CIP concentration, the initial CIP concentration varied at values of 1.6, 5.9, and 10.3 mg/L (contact time = 24 h, pH unadjusted). The *t*-test was performed to study the significant differences in removal efficiencies after preconditioning. The adsorption capacity of MPB at each CIP concentration was calculated using the following equation:

1 $$q_\mathrm{e}\;=\;\frac{\left(C_{0\;\;}-\;C_e\right)\;V}m$$

In Eq. (1), *q*_e_ represents the antibiotic adsorption capacity (mg/g), *C*_0_ is the initial concentration of antibiotic (mg/L), *C*_e_ is the final concentration of the antibiotic (mg/L), *V* stands for the volume of the antibiotic solution (L), and *m* refers to the weight of the biosorbent (g).

### Characterisation of microbiomes from biosorbent preconditioned with hospital wastewater MBR effluent

To obtain insight into the bacteria colonising the tested biosorbent materials preconditioned with hospital wastewater MBR effluent, DNA was extracted and 16S rRNA gene amplicon sequencing was performed. In addition to the wastewater effluent-preconditioned biosorbents described in Sect. “Preconditioning MPB biosorbent with bacteria or hospital wastewater effluent”, an MPB (1 g/L; *n* = 1) control treatment was established by preconditioning the biosorbent with sterile Milli-Q water for 3 h, 24 h, 48 h, and 1 week, to evaluate background levels in the biosorbent material.

DNA was extracted from preconditioned biosorbent materials (500 mg) using the Fast DNA Spin Kit for Soil, following the manufacturer’s instructions (MP Biomedicals). DNA quality and quantity were checked using a Nanodrop ND-1000 spectrophotometer. DNA concentrations after 3 h were very low in the wastewater-exposed samples and could not be used for amplicon sequencing. Conversely, for the control samples, DNA concentrations were very low after 48 h and 1 week of exposure, and amplicon sequencing was therefore only conducted for the two first exposure time periods (3 h and 24 h). The primers 515 F (5′-GTGCCAGCMGCCGCGGTAA-3′) and 806R (5′GGACTACHVGGGTWTCTAAT-3′) were used to amplify the V4 region of the bacterial 16S rRNA gene (Walters et al. 2015). A small-fragment library was constructed, followed by paired-end sequencing using the Illumina MiSeq platform (StarSEQ GmbH, Mainz, Germany). The DADA2 pipeline (Callahan et al. 2016) in R version 4.1 was employed to process the 16S rRNA gene amplicon sequences. Forward and reverse reads were filtered and trimmed using DADA2’s default settings, with the exception of truncLen set to 280 for forward reads and 220 for reverse reads. The paired-end reads were then merged, and an amplicon sequence variant (ASV) database was created using DADA2’s core sample inference algorithm and trained error models, followed by chimera removal. Taxonomic assignment of the ASVs was performed using the Silva 138.1 prokaryotic SSU taxonomic training data (Quast et al. 2013). For downstream analysis, ASVs identified as mitochondrial or chloroplast DNA were removed, resulting in a total of 1020 ASVs.

The ZymoBIOMICS™ Microbial Community DNA Standard (Zymo Research Corp., Irvine, CA, USA) was used to verify the 16S rRNA gene sequencing results. The Microbial Community DNA Standard exhibited a spurious ASV proportion of just 0.024%, which is notably below the previously recommended threshold of 0.25% (Reitmeier et al. 2021). Furthermore, the abundance variations between the theoretical and observed bacterial communities were mostly under 15%, meeting the standards established by ZymoBIOMICS Microbial Standards (Table S1).

#### Statistical analysis of bacterial community data

R version 4.1 was employed for generating graphics and performing all statistical analyses to characterise the bacterial community from 16S rRNA amplicon sequencing data. The bacterial community composition was analysed using the “microeco” (v0.13.0), “ampvis2” (v2.7.31), and “ggplot2” (v3.4.0) R packages (Andersen et al. 2018; Liu et al. 2021; Wickham 2016). Alpha diversity of the samples was illustrated using Chao1 and Shannon indices, with statistical differences between treatment groups evaluated via the Wilcoxon rank-sum test. Beta diversity was visualised using principal coordinates analysis (PCoA) based on Bray-Curtis dissimilarity metrics. Differences in bacterial community composition among treatment groups were further explored using multivariate analysis of variance (MANOVA).

## Results and discussion

### Impact of exposure of magnetite-pine bark to bacteria and hospital wastewater effluent on CIP removal efficiency

**Fig. 1:**
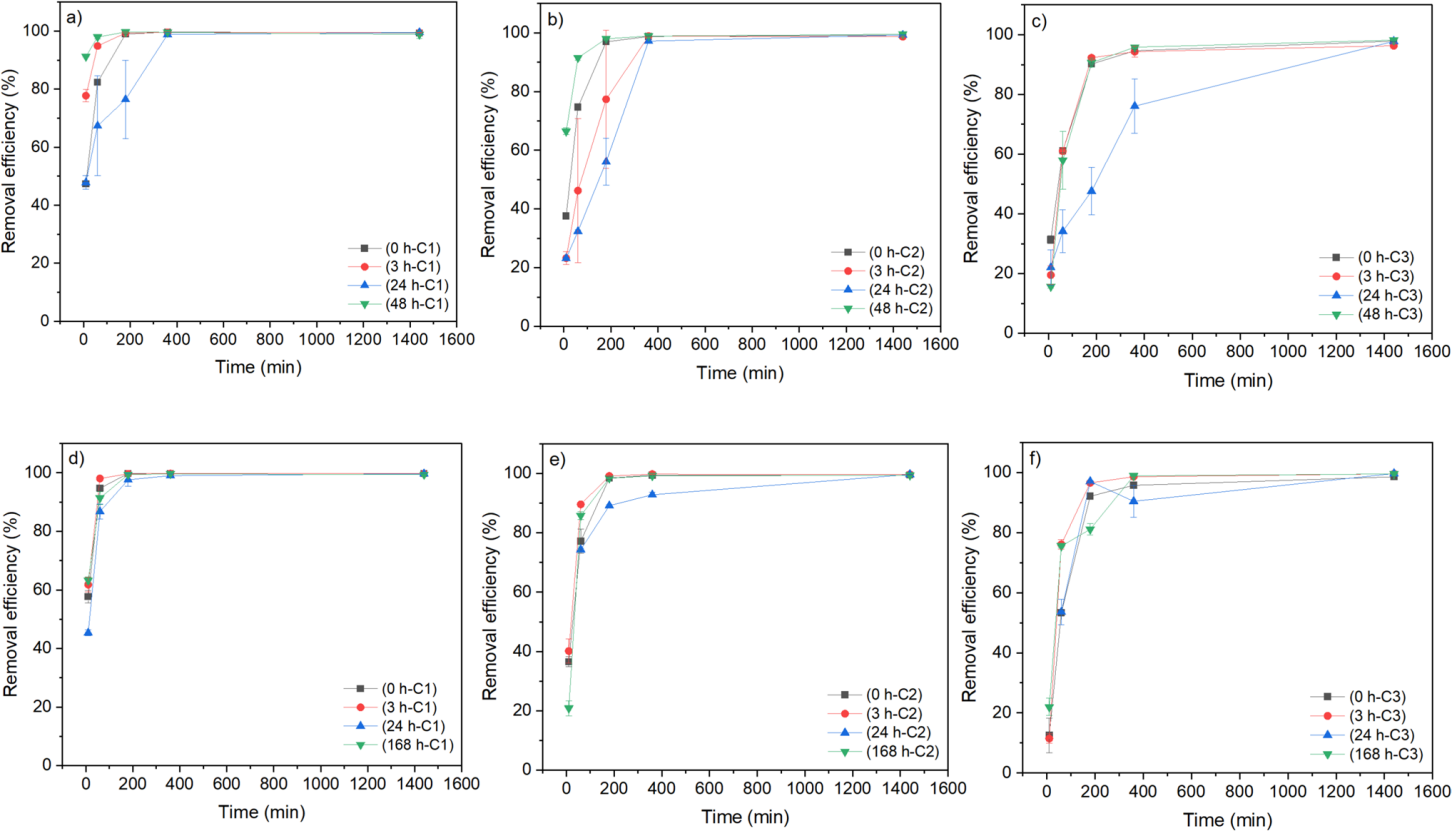
Effect of preconditioning time with **a**–**c** *Aeromonas rivipollensis* bacteria strain (0 h (control), 3 h, 24 h, and 48 h) and **d**–**f** hospital wastewater effluent with complex microbial community (0 h (control), 3 h, 24 h, and 168 h) on the removal efficiency of ciprofloxacin over magnetite-pine bark (biosorbent dosage 1 g/L, initial antibiotic concentration C1: 1.6 mg/L, C2: 5.9 mg/L, and C3: 10.3 mg/L, pH: 4.8–5.8)

The CFU counts showed that, after 1 h incubation, plates with MPB exposed to *Aeromonas* showed fewer CFUs compared to plates with bacteria only, whereas after 3 h, no notable difference was observed between the two treatments, suggesting a transient antimicrobial effect of MPB. There were more than 200,000 CFU/mL in all samples. To evaluate the impact of preconditioning time with pure culture bacteria and hospital wastewater effluent, the preconditioned MPB was tested at different exposure timepoints (Fig. 1). Preconditioning affected adsorption efficiency at lower contact times (10–360 min) and reached almost the same removal efficiency (98.2–99.6%) at 24-h contact time in all cases. The observed initial resistance is mainly associated with bacterial preconditioning and is not evident in the MPB–CIP system without bacterial preconditioning (0 h). In the absence of bacteria, adsorption proceeds rapidly. In contrast, preconditioned MPB especially at 24 h shows slower initial uptake, likely due to increased site occupation and diffusion resistance caused by biofilm development during the bacterial growth. At the lowest CIP concentration (C1 = 1.6 mg/L), preconditioning with bacteria (Fig. 1a) at 24 h had lower removal efficiency between 10 and 360 min contact times, but the 3-h and 48-h preconditioning timepoints resulted in higher removal efficiency with a 15.1–90.9% increase at 10- to 60-min contact times. Besides, preconditioning with wastewater effluent (Fig. 1d) at 24 h decreased removal efficiency slightly, with fewer significant changes compared to preconditioning with bacteria (Fig. 1a). At CIP concentration of C2: 5.9 mg/L, preconditioning with bacteria (Fig. 1b) at 48 h increased adsorption efficiency at 10-min and 60-min contact times (by 76.8% and 22.5%, respectively), while preconditioning at 3 h and 24 h reduced it at 360-min contact time. Besides, preconditioning with wastewater effluent (Fig. 1e) at 24 h reduced removal efficiency slightly at 24-h contact time.

At the highest CIP concentration (C3 = 10.3 mg/L), preconditioning with bacteria (Fig. 1c) at all three timepoints initially slightly decreased the adsorption efficiency at 10-min contact time, but only preconditioning at 24 h decreased efficiency with longer contact times until 24 h was reached. On the other hand, preconditioning with wastewater effluent (Fig. 1f) at 3 h increased efficiency at 24-h contact time. Preconditioning with wastewater effluent at 24 h had no significant impact, while preconditioning at the longest timepoint (168 h) increased efficiency except for 180-min contact time.

The results demonstrate that preconditioning with bacteria and wastewater effluent at 24-h colonisation time mostly decreased the adsorption efficiency by 18.2–56.6% and 3.7–21.3%, respectively, but other colonisation times increased adsorption efficiency by 15.1–90.9% and 3.3–91.5%, respectively, with some exceptions. In addition, there was no significant difference between the removal efficiencies statistically (*t*-test, *p* < 0.05, two-tailed). These findings suggest that the microbiome associated with the biosorbent can alter its surface properties and ability to remove micropollutants (Rivera-Utrilla et al. 2003; Rivera‐Utrilla et al., 2001), which can either enhance or hinder CIP adsorption depending on exposure time, CIP concentration, and contact time. The surface alteration of the biosorbent can be caused by the accumulation of microbial byproducts on the surface or prolonged changes to the surface after exposure to bacteria (Rivera-Utrilla et al. 2003; Wilcox et al. 1983). The results of the present study provide a detailed understanding regarding the impacts of the time of bacterial exposure on enhancing or reducing adsorption efficiency.

The adsorption process consists of three key stages: first, the external mass transfer of the adsorbate from the bulk solution to the adsorbent’s outer surface; second, the internal diffusion of the adsorbate towards the active sites within the adsorbent; and finally, the adsorption onto these sites. To describe the adsorption process, experimental adsorption data of CIP was fitted with non-linear pseudo-first-order (PFO) and non-linear pseudo-second-order (PSO) models (Figure S1), which have been elaborated in the supplementary material, and the parameters of the models are summarised in Table S2. Depending on preconditioning type and time, and initial CIP concentration, both PFO and PSO fitted the experimental adsorption data of CIP when the correlation coefficients (*R*^2^) were closer to 1 and reduced chi-square (*χ*^2^) was lower in comparison to another model. Without preconditioning with bacteria or wastewater effluent (Figure S1 a), the PFO model fitted well with the experimental data, with the *R*^2^ closer to 1 than those of PSO. At 3-h preconditioning with bacteria or wastewater (Figure S1 b & e), the PFO model fitted well with the experimental data, except for preconditioning with bacteria at the lowest CIP concentration (C1: 1.6 mg/L), which PSO fitted better. At 24-h preconditioning with both bacteria and wastewater (Figure S1 c & f), PSO fitted well at the lowest CIP concentration (C1: 1.6 mg/L) and at the highest CIP concentration (C3: 10.3 mg/L) with preconditioning with bacteria and with wastewater at C2: 5.9 mg/L; otherwise, PFO exhibited better fitting. At 48-h preconditioning with bacteria (Figure S1d), PSO fitted well with lower concentrations of CIP (C1: 1.6 mg/L and C2: 5.9 mg/L), while PFO presented a better fit with C3: 10.3 mg/L. On the other hand, PFO fitted with the longest preconditioning time with wastewater (1 week) (Figure S1 g) and all concentrations of CIP. Overall, the differences in fitted models based on preconditioning time indicate that microbial colonisation and exposure time can alter the adsorption mechanism, shifting it from primarily PFO to PSO depending on the conditions. A better PFO fit for most cases suggests that adsorption is probably governed primarily by physical surface interactions and possibly external mass transfer. On the other hand, better fits with PSO, especially at lower concentrations and typically 24-h exposure times, may imply chemical interactions resulting from surface modification by microbial biofilms, which caused a reduction of adsorption efficiency as discussed above in Sect. “Impact of exposure of magnetite-pine bark to bacteria and hospital wastewater effluent on CIP removal efficiency”. Previous studies have shown that CIP adsorption has been fitted with the PSO model (Al-sareji et al. 2024; Farzinmanesh et al. 2024; Oliveira et al. 2023) and also with the PFO model (Khan et al. 2024). The results obtained from the present research, in line with earlier findings, show that adsorption behaviour depends strongly on the specific system conditions. Importantly, these findings demonstrate that, despite such mechanistic differences, MPB biosorbent consistently achieves effective CIP removal efficiency, underscoring its robustness as a sustainable option for water treatment applications.

### CIP adsorption capacity at different concentrations

The achieved equilibrium adsorption capacity of MPB for CIP at the tested conditions was 1.6, 5.9, and 10.1 or 10.2 mg/g after preconditioning with bacteria or wastewater effluent. This suggests that MPB could adsorb CIP almost completely after preconditioning with both bacteria and wastewater. It was shown earlier that banana peel biochars had 22.0–23.4 mg/g experimental adsorption capacity of CIP from synthetic solution (Patel et al. 2021), and activated carbon from Cupuaçu bark yielded equilibrium adsorption capacity of 5.9 mg/g for CIP from aqueous solution (do Nascimento et al. 2024). Besides, maximum adsorption capacity for CIP from synthetic solution onto magnetite-imprinted chitosan polymer nanocomposites was 142.8 mg/g (Rasoulzadeh et al. 2019). In the previous work (Mohammadzadeh and Leiviskä, 2023), MPB showed higher equilibrium adsorption capacity of levofloxacin (153.0 mg/g) and trimethoprim (184.1 mg/g) in batch mode. Therefore, it is expected that CIP adsorption capacity could have been similarly high when testing various higher initial concentrations.

**Fig. 2:**
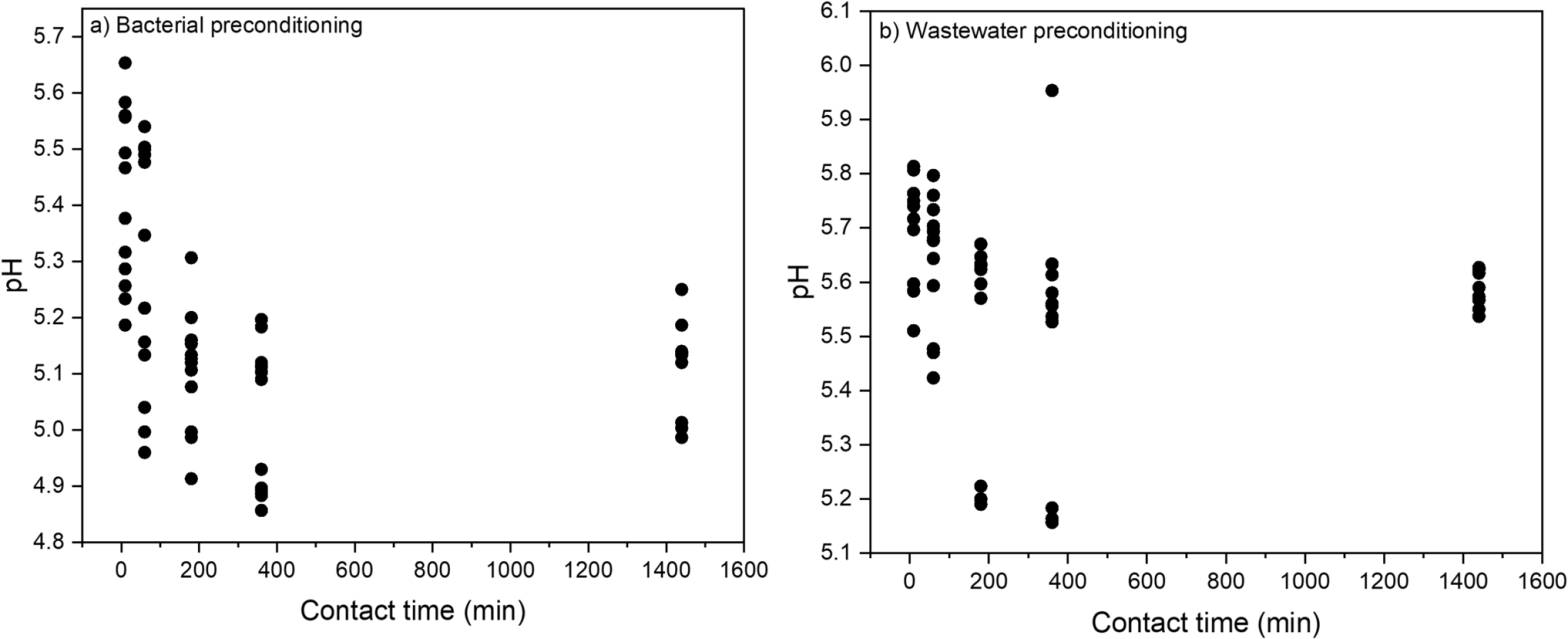
The final pH of CIP solutions at different contact times (10, 60, 180, 360, & 1440 min) with **a** bacterial and **b** wastewater preconditioning

After batch shaking, the final pH of CIP solutions at different contact times was almost between 4.8 and 5.8 after preconditioning the biosorbent with bacteria (Fig. 2a), and it was mostly 5.1–5.8 after preconditioning with wastewater effluent (Fig. 2b). The pH values varied based on different contact times with CIP solution, where at the same initial CIP concentration and the same exposure time, by increasing the contact time, the pH had a slightly decreased pattern in most cases. The pH_pzc_ (pH at point of zero charge) of MPB was 6.5 based on the previous study (Mohammadzadeh and Leiviskä, 2023). Below the 6.5 pH value, MPB possesses a positive charge, and above 6.5, it has a negative surface charge. CIP with pK_a1_ = 5.9 and pK_a2_ = 8.9 (Igwegbe et al. 2021) can be ionised positively below pH 5.9, negatively above pH 8.9, and neutral between these two pH values. Therefore, since the pH of the CIP solution after adsorption was 4.8–5.8, the electrostatic repulsion may inhibit the adsorption. This suggests that electrostatic interactions are not the primary mechanism in this system and that other mechanisms play a more dominant role. Furthermore, it is likely that preconditioning with bacteria and wastewater effluent might have reduced the pH_pzc_.

**Fig. 3:**
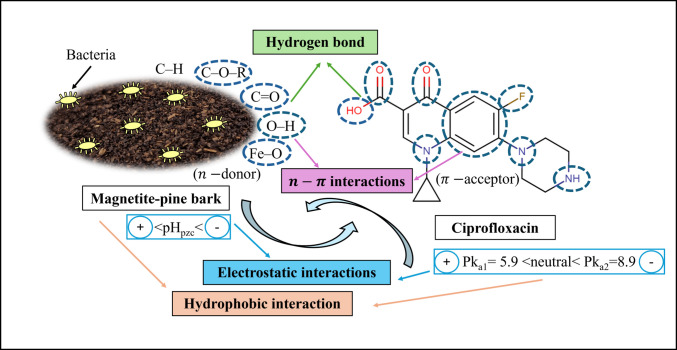
Assumptions on the possible adsorption mechanisms of CIP on magnetite-pine bark

Based on the previous FTIR analysis of MPB (Mohammadzadeh and Leiviskä, 2023), the presence of phenolic O–H, aliphatic C–H, carbonyl C=O, C–O–R, and Fe–O groups has been confirmed, and possible assumptions on the adsorption mechanisms of CIP on MPB were proposed (Fig. 3). Hydrogen bonding may play a crucial role in the adsorption mechanism of CIP, given its nature as a polar organic molecule (Laabd et al. 2021). It is expected that the hydroxyl (O–H) and carbonyl (C=O) groups of CIP will bond with the oxygen containing groups of C=O, C–O–R, Fe–O, and O–H on the MPB, which was also a dominant expectation for levofloxacin adsorption on MPB, since a decrease in the intensity of O–H functional groups peak was observed in the FTIR analysis after adsorption (Mohammadzadeh and Leiviskä, 2023). The hydrogen atom in amine (–NH) and O–H groups of CIP will tend to bond with C=O, C–O–R, Fe–O, and O–H groups on the MPB. Besides, fluorine, oxygen, and nitrogen-containing groups of CIP, which are hydrogen bond acceptors, will bond with the O–H groups on the MPB. The benzene ring in the structure of CIP can act as a hydrogen bond acceptor to form bonding with the O–H groups on the MPB (Afzal et al. 2019). Therefore, multiple hydrogen bonding possibilities will most probably play an important role in the adsorption of CIP on MPB. The n-π electron donor-acceptor interactions between the aromatic ring of CIP (acting as a π-acceptor due to the electron-withdrawing effect of the fluorine atom) and the oxygen-containing groups of the MPB (acting as n-donors) may also contribute to the adsorption mechanism of CIP, which has also been suggested for adsorption of CIP onto a Hydroxyapatite@Montmorillonite hybrid composite (Laabd et al. 2021). It was reported by other research (Peng et al. 2016) that neutral CIP showed higher hydrophobicity compared to positively and negatively charged CIP, and the hydrophobic interaction was the dominant mechanism in adsorption of CIP on graphitic ordered mesoporous carbons. Therefore, it is probable that hydrophobic interaction can have a role in the adsorption of CIP on MPB, since preconditioning with bacteria and wastewater effluent most likely has increased hydrophobicity and impacted the adsorption.

### Composition of MPB biosorbent-associated bacterial communities following preconditioning with hospital wastewater MBR effluent

**Fig. 4:**
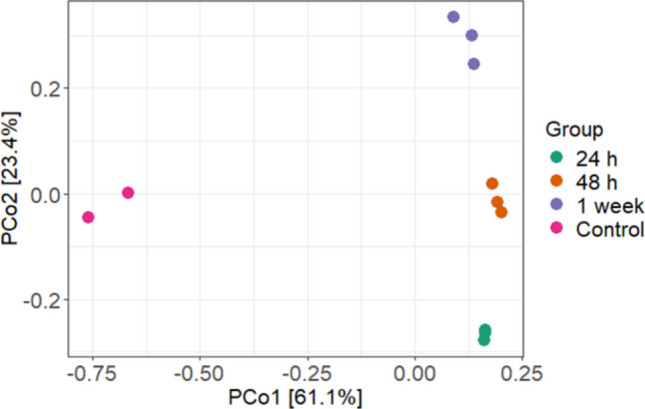
Principal coordinates analysis (PCoA) plot illustrating the beta diversity of microbial communities associated with magnetite-pine bark biosorbents exposed to hospital wastewater (1 g/L) over three distinct colonisation periods: 24 h (*n* = 3), 48 h (*n* = 3), and 1 week (*n* = 3). The Bray-Curtis dissimilarity index was used to calculate the differences in microbial community composition, with each point representing a sample in the ordination space. Additionally, control samples with magnetite-pine bark biosorbents (1 g/L) were analysed after 3 h (*n* = 1) and 24 h (*n* = 1) of exposure to sterile water, serving as a baseline for comparison

After quality filtering, the MPB biosorbent samples (*n* = 11) yielded a total of 1,598,549 paired-end 250-bp sequences, ranging from 99,170 to 188,428 sequences per sample. These sequences were clustered into 1020 ASVs, which were assigned to 21 different phyla and 296 genera. The diversity of MPB biosorbent-associated bacterial communities increased progressively with biosorbent preconditioning time; that is, following exposure to hospital wastewater MBR effluent for 1, 2, or 7 days (Figure S2). Preconditioning time also affected the bacterial community composition (Figure S3; Fig. 4). Biosorbents preconditioned with wastewater effluent contained microbiomes that were distinctly different from those in control biosorbents exposed to sterile water (Fig. 4). This indicates that bacteria from the wastewater actively colonised the biosorbent material, whereas the distinct microbiomes in control biosorbents were most likely derived from the bacteria initially present within the biosorbent matrix. The dominance of endospore-forming taxonomic groups in biosorbent preconditioned with sterile water is consistent with this interpretation.

**Fig. 5:**
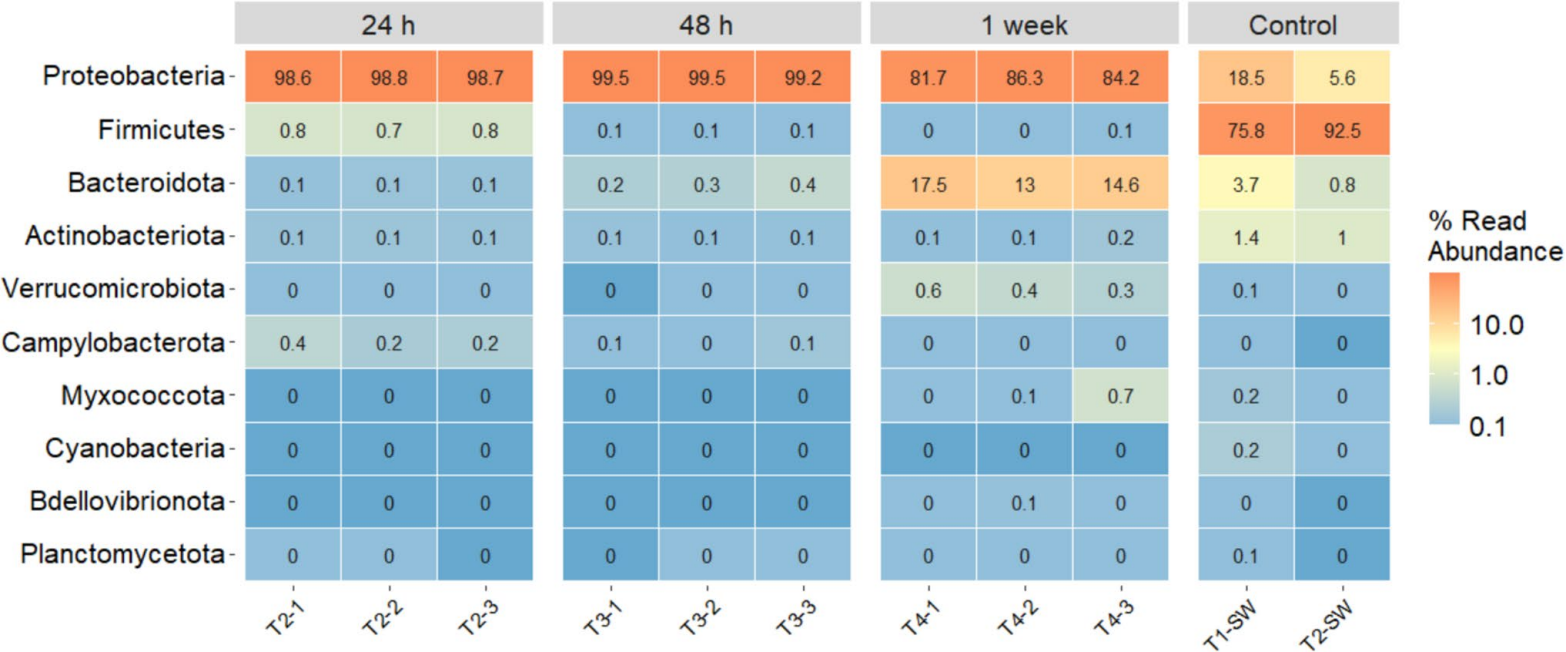
Heatmap illustrating the relative abundance of the 10 most abundant microbial phyla identified in magnetite-pine bark biosorbent after exposure to hospital wastewater (1 g/L) over three distinct colonisation periods: T2: 24 h (*n* = 3), T3: 48 h (*n* = 3), and T4: 1 week (*n* = 3). Additionally, control samples with magnetite-pine bark biosorbents (1 g/L) were analysed after T1-SW: 3 h (*n* = 1) and T1-SW: 24 h (*n* = 1) of exposure to sterile water

Further analysis revealed that the bacteria present on the wastewater-exposed biosorbents were predominantly composed of the phylum *Proteobacteria*, with relative abundances ranging from 81.7 to 99.5% (Fig. 5). In contrast, *Proteobacteria* were much less abundant in control samples exposed to sterile water (18.5% and 5.6%), suggesting they primarily originated from the wastewater. Control samples were, in contrast, dominated by *Firmicutes* (75.8% and 92.5%), indicating that this phylum may be inherent to or associated with the biosorbent, although it did not persist after wastewater exposure. *Proteobacteria* have been reported as the dominant phylum in different wastewater treatment plants, including in full-scale MBR systems and rural MBR-based plants (Feng et al. 2022; Li et al. 2022; Wang et al. 2025). A pilot-scale study of an anaerobic membrane bioreactor showed that *Firmicutes* and *Proteobacteria* were among the major bacterial phyla (Kong et al. 2022).

At the genus level, *Pseudomonas* was the most abundant genus in biosorbents exposed to wastewater, with relative abundances of 27.5–25.2% at 24 h, 21.9–15.9% at 48 h, and 10.6–14.6% at 1 week, showing a decline over time (Fig. 5). In contrast, *Geobacillus*, which is a gram-positive genus in the *Bacillaceae* family, was dominant in the control samples exposed to sterile water (64.7% and 65.2%) (Fig. 5). The genus *Pseudomonas* (family *Pseudomonadaceae*) ranks among the largest genera of gram-negative bacteria (Girard et al. 2021). *Pseudomonas* species naturally inhabit soil, water, and various other environments (Ruiz et al. 2004). *Pseudomonas* are known to carry multiple antibiotic resistance genes (Fariñas and Martínez-Martínez 2013). The genus *Geobacillus* is commonly found in nature and is particularly prevalent in extreme environments such as cool soils, hot springs, hydrothermal vents, deep-sea trenches, hay compost, and dairy processing facilities (Khaswal et al. 2022). Therefore, its presence in MPB can be attributed to the natural structure of pine bark as a forestry residue.

In addition to *Pseudomonas* as the most abundant genus, several other abundant genera were also identified in MPB after exposure to wastewater effluent (Fig. 5). *Aquabacterium* and *Acidovorax* (family *Comamonadaceae*) were consistently abundant, suggesting potential roles in antibiotic degradation. *Aquabacterium commune* was shown to be a highly abundant species in drinking water biofilms originating from distribution systems in several European cities (Kalmbach et al. 2000). *Aquabacterium* was shown to be capable of degrading sulfadiazine and microplastics (Chen et al. 2024). Previous studies revealed that *Acidovorax* was highly abundant in activated sludge from municipal wastewater treatment plants (Schulze et al. 1999), and it also contributed notably to the removal of sulfamethoxazole, COD, and sulphonamide-resistance gene (Yu et al. 2025). These results show that different bacterial genera dominated at different colonisation times. For example, *Curvibacter*, *Dechloromonas*, and *Rheinheimera* were more abundant at 24 h, while *Flavobacterium*, *Cellvibrio*, and *Rhizobia* became more abundant later (at 1 week). Genera from the *Comamonadaceae* family, such as *Aquabacterium* and *Acidovorax*, remained consistently abundant across timepoints, suggesting they may play key roles in the degradation of sulphonamide antibiotics, as discussed above.

Species such as *Pseudomonas putida*, *Geobacillus thermocatenulatus*, *Bacillus badius*, *Escherichia coli*, *Bacillus cereus*, *Rhodococcus rhodochrous*, *Exiguobacterium* sp. RD3, and *Phanerochaete chrysosporium* are known to degrade pharmaceuticals and their metabolites in aquatic environments (Narayanan et al. 2022). It was reported that a thermophilic bacterium closely related to *Geobacillus thermoleovorans*, isolated from pharmaceutical sludge, degraded sulfamethazine antibiotic effectively at 70 °C (Pan et al. 2017). It was also shown that *Pseudomonas* effectively degraded sulfamethoxazole through multiple metabolic pathways, without carrying detectable sulfonamide resistance genes (Liu et al. 2022). Endospore-forming bacteria, which are highly resilient, were detected in the control samples but not after adsorption.

## Conclusions

Preconditioning MPB with the *Aeromonas* bacteria strain and hospital wastewater effluent with a complex microbial community impacted CIP removal efficiency in a contrasting pattern based on the different exposure time of the biosorbent to bacteria or wastewater effluent, initial CIP concentration, and contact times. Preconditioning for 24 h generally decreased adsorption efficiency (bacteria: 18.2–56.6%, wastewater: 3.7–21.3%), while other colonisation times mostly increased it (bacteria: 15.1–90.9%, wastewater: 3.3–91.5%), with some exceptions. The comparison of *Aeromonas* with the adhered bacteria after exposure of MPB to wastewater effluent revealed that *Pseudomonas* was the most abundant bacteria adhered to the biosorbent. Several other abundant genera were identified in MPB after exposure to wastewater, of which the *Comamonadaceae* family was consistently abundant over time. On the other hand, *Geobacillus* was dominant only in the MPB biosorbent, showing it originated from MPB. Further research must be conducted to propose a possible removal mechanism for CIP in the MPB system after exposure to bacteria.

## Supplementary Information

Below is the link to the electronic supplementary material.

ESM 1 (DOCX 506 KB)

## Data Availability

The data supporting the findings of this study are available within the supplementary materials.
