# Supplementary material for: Ciprofloxacin adsorption to magnetite-pine bark biosorbents as affected by preconditioning with distinct microbiomes
Source: Environ Sci Pollut Res Int. 2026 Apr 30;33(16):7549–60. doi: 10.1007/s11356-026-37778-w (PMC13190525; doi:10.1007/s11356-026-37778-w)
Supplement: Supplementary file 1 — (DOCX 506 KB) [file 11356_2026_37778_MOESM1_ESM.docx]

**Ciprofloxacin adsorption to magnetite-pine bark biosorbents as affected by preconditioning with distinct microbiomes**

Mahdiyeh Mohammadzadeh ^a*^, Simon Bo Lassen ^b^, Mia Kristine Staal Jensen ^b,c^, Bjarne Westergaard Strobel ^b^, Kristian Koefoed Brandt ^b^, Tiina Leiviskä ^a^

^a^ Chemical Process Engineering, University of Oulu, P.O. Box 4300, Oulu FIN-90014, Finland

^b^ Department of Plant and Environmental Sciences, University of Copenhagen, Thorvaldsensvej 40, DK-1871 Frederiksberg C, Denmark

^c^ Sino-Danish Center for Education and Research (SDC), Beijing 100049, China

* Corresponding author (Mahdiyeh.Mohammadzadeh@oulu.fi)

**Supplementary Material**

**Table S1: Comparisons were made between a MOCK community for 16S sequencing, comprising genomic DNA from eight bacterial species (ZymoBIOMICS™ Microbial Community DNA Standard). The table presents the theoretical 16S rRNA gene composition (%), the observed number of Amplicon Sequence Variants (ASVs), their respective abundances (%), and the variations in abundance (%) between the theoretical and observed bacterial compositions. Direct read-mapping against reference 16S rRNA sequences showed a generally cohesive relationship between the theoretical and observed bacterial community compositions, with variations typically under 15%, as commonly accepted for ZymoBIOMICS Microbial Standards. Notably, Pseudomonas aeruginosa was overestimated (43.5%), while Lactobacillus fermentum and Bacillus subtilis were underestimated (-19.4% and -20.2%, respectively).**

| Species | Theoretical composition (%) 16S rRNA gene | ASV counts | ASV abundance (%) | Abundance variation (%) |
| --- | --- | --- | --- | --- |
| *Pseudomonas aeruginosa* | 4.2 | 4,925 | 6.0 | 43.5 |
| *Escherichia coli* | 10.1 | 9,456 | 11.6 | 14.6 |
| *Salmonella enterica* | 10.4 | 9,122 | 11.2 | 7.3 |
| *Lactobacillus fermentum* | 18.4 | 12,113 | 14.8 | -19.4 |
| *Enterococcus faecalis* | 9.9 | 8,790 | 10.8 | 8.7 |
| *Staphylococcus aureus* | 15.5 | 14,433 | 17.7 | 14.0 |
| *Listeria monocytogenes* | 14.1 | 11,527 | 14.1 | 0.0 |
| *Bacillus subtilis* | 17.4 | 11,346 | 13.9 | -20.2 |

**Kinetic modelling:**

The adsorption kinetics of CIP on magnetite-pine bark was investigated by fitting the experimental data with non-linear pseudo-first-order (PFO) (Lagergren, 1898) and non-linear pseudo-second-order (PSO) (Blanchard et al., 1984) models. The non-linear pseudo-first-order equation is expressed as:

$q_{t}$=$q_{e}$ (1- $e^{-k_{1}t}$) (1)

where q_t_ and q_e_ represent the adsorption capacity (mg/g) at time t (min) and equilibrium, respectively, and k_1_ (1/min) is the pseudo-first-order rate constant.

The non-linear pseudo-second-order equation is expressed as:

$q_{t}$ = $\frac{q_{e}^{2} k_{2}t}{1+k_{2}q_{e}t}$ (2)

where q_t_ and q_e_ are the adsorption capacity (mg/g) at any time t (min) and equilibrium, respectively, and k_2_ (g/mg min) is the pseudo-second-order rate constant.

**Table S2. Parameters of the pseudo-first-order (PFO) and pseudo-second-order (PSO) models. (Initial antibiotic concentration C1: 1.6 mg/L, C2: 5.9 mg/L and C3: 10.3 mg/L) (0 h-48 h: preconditioning time with bacteria in the first part of the table, and 0 h-168 h: preconditioning time with wastewater effluent in the second part of the table).**

| Models | Parameters | 0 h-C1 | 0 h-C2 | 0 h-C3 | 3 h-C1 | 3 h-C2 | 3 h-C3 | 3 h-C1 | 24 h-C2 | 24 h-C3 | 48 h-C1 | 48 h-C2 | 48 h-C3 |
| --- | --- | --- | --- | --- | --- | --- | --- | --- | --- | --- | --- | --- | --- |
| Pseudo-first-order  (PFO) | k_1_ (1/min) | 0.056 | 0.043 | 0.014 | 0.152 | 0.026 | 0.017 | 0.065 | 0.009 | 0.005 | 0.248 | 0.109 | 0.017 |
|  | q_e_ (mg/g) | 1.564 | 5.887 | 10.016 | 1.563 | 5.853 | 9.875 | 1.563 | 5.885 | 10.043 | 1.565 | 5.893 | 10.041 |
|  | R^2^ | 0.881 | 0.976 | 0.966 | 0.810 | 0.998 | 0.997 | 0.985 | 0.968 | 0.958 | 0.991 | 0.831 | 0.999 |
|  | χ^2^ | 46.513 | 22.554 | 27.226 | 8.512 | 0.932 | 1.184 | 2.313 | 572.139 | 3.998 | 2.559 | 53.494 | 6.972 |
| Pseudo-second-order  (PSO) | k_2_ (g/mg×min) | 0.217 | 0.017 | 0.003 | 0.267 | 0.010 | 0.003 | 0.060 | 0.002 | 5.977 | 0.664 | 0.037 | 0.002 |
|  | q_e_ (mg/g) | 1.575 | 5.941 | 10.338 | 1.566 | 5.995 | 10.464 | 1.579 | 6.199 | 11.084 | 1.572 | 5.921 | 10.453 |
|  | R^2^ | 0.518 | 0.883 | 0.887 | 0.880 | 0.905 | 0.929 | 0.986 | 0.965 | 0.969 | 0.995 | 0.981 | 0.992 |
|  | χ^2^ | 188.335 | 111.383 | 90.128 | 5.389 | 35.881 | 28.319 | 2.184 | 614.767 | 2.954 | 1.400 | 6.023 | 64.787 |
| Models | Parameters | 0 h-C1 | 0 h-C2 | 0 h-C3 | 3 h-C1 | 3 h-C2 | 3 h-C3 | 3 h-C1 | 24 h-C2 | 24 h-C3 | 168 h-C1 | 168 h-C2 | 168 h-C3 |
| Pseudo-first-order  (PFO) | k_1_ (1/min) | 0.056 | 0.043 | 0.014 | 0.095 | 0.038 | 0.020 | 0.060 | 0.020 | 0.020 | 0.101 | 0.031 | 0.024 |
|  | q_e_ (mg/g) | 1.564 | 5.887 | 10.016 | 1.567 | 5.909 | 10.215 | 1.567 | 5.904 | 10.226 | 1.563 | 5.890 | 10.200 |
|  | R^2^ | 0.881 | 0.976 | 0.966 | 0.985 | 0.969 | 0.984 | 0.994 | 0.818 | 0.926 | 0.984 | 0.986 | 0.950 |
|  | χ^2^ | 46.513 | 22.554 | 27.226 | 1.195 | 4.529 | 18.699 | 6.137 | 301.531 | 8.599 | 4.771 | 5.114 | 40.890 |
| Pseudo-second-order  (PSO) | k_2_ (g/mg×min) | 0.217 | 0.017 | 0.003 | 0.300 | 0.078 | 0.008 | 0.054 | 0.007 | 0.015 | 0.130 | 0.026 | 0.008 |
|  | q_e_ (mg/g) | 1.575 | 5.941 | 10.338 | 1.577 | 5.925 | 10.311 | 1.580 | 6.000 | 10.272 | 1.583 | 5.919 | 10.333 |
|  | R^2^ | 0.518 | 0.883 | 0.887 | 0.519 | 0.328 | 0.811 | 0.996 | 0.996 | 0.661 | 0.903 | 0.649 | 0.744 |
|  | χ^2^ | 188.335 | 111.383 | 90.128 | 37.873 | 97.251 | 221.979 | 4.204 | 7.411 | 39.336 | 29.717 | 129.708 | 209.296 |


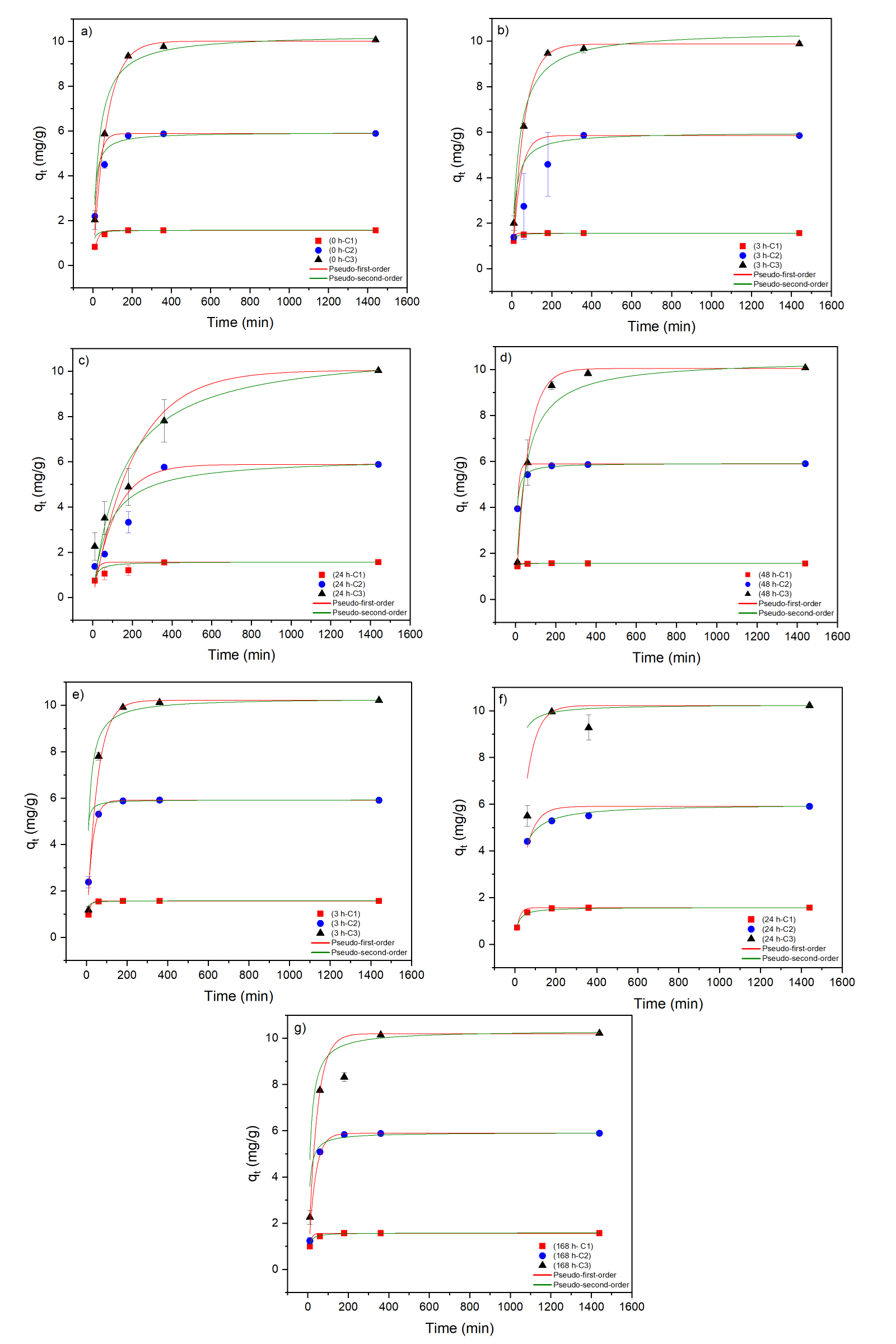


**Figure S1. Non-linear pseudo-first-order and pseudo-second-order models and experimental kinetics for the adsorption of CIP over magnetite-pine bark (biosorbent dosage 1 g/L****, initial antibiotic concentration C1: 1.6 mg/L, C2: 5.9 mg/L and C3: 10.3 mg/L, pH: 4.8-5.8, without pH adjustment) (0 h-48 h: preconditioning time with bacteria: (a-d), and 0 h-168 h: preconditioning time with wastewater effluent: (a & e-g)).**


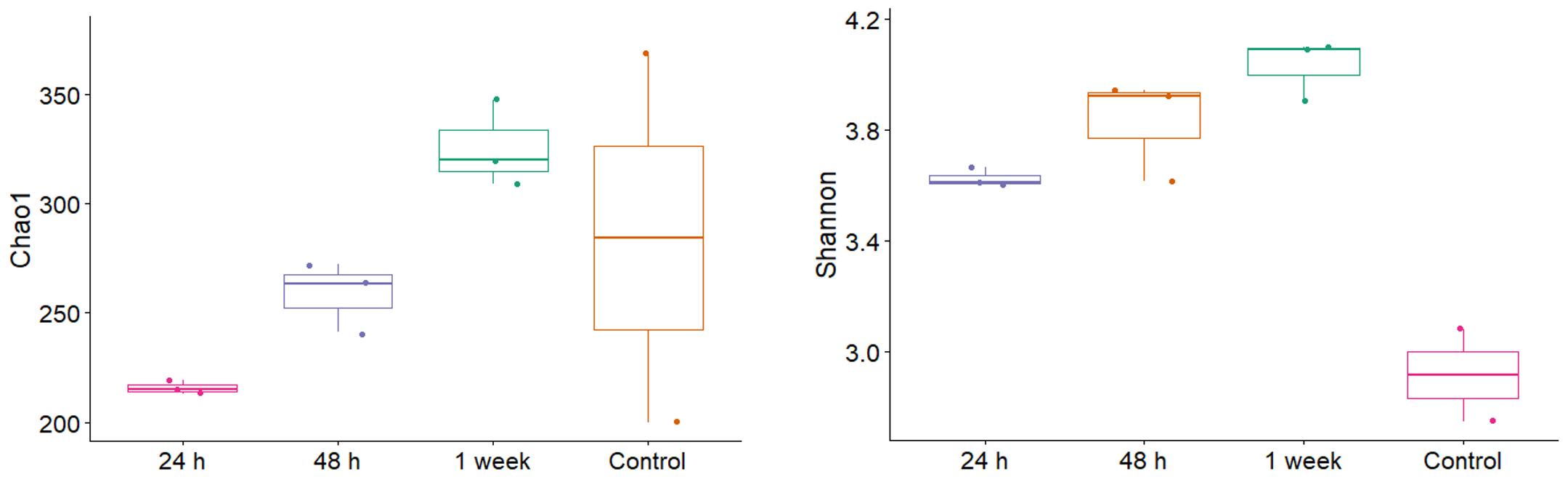


**Figure S2. Boxplots illustrating the microbial diversity indices, Chao1 and Shannon, for magnetite-pine bark biosorbents exposed to hospital wastewater (1g/L) across three distinct colonisation periods: 24 hours (n=3), 48 hours (n=3), and 1 week (n=3). The Chao1 index estimates species richness, reflecting the number of different species present, while the Shannon index accounts for both abundance and evenness of the species. Additionally, control samples with magnetite-pine bark biosorbent (1g/L) were analysed after 3 h (n=1) and 24 h (n=1) of exposure to sterile water to serve as a baseline for comparison.**


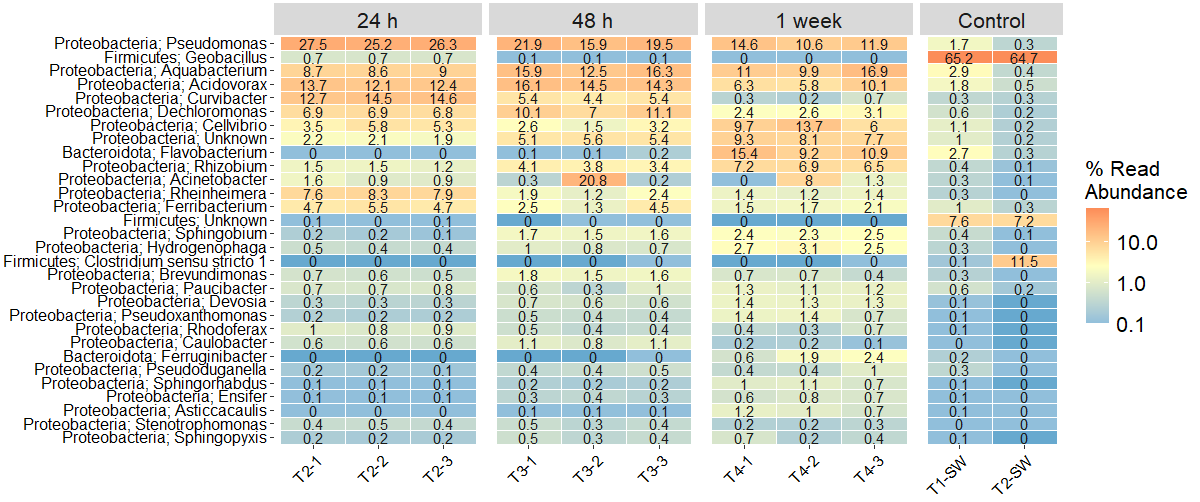


Figure S3: Heatmap illustrating the relative abundance of the 30 most abundant bacterial genera identified in magnetite-pine bark biosorbents after exposure to hospital wastewater (1g/L) over three distinct colonisation periods: T2: 24 h (n=3), T3: 48 h (n=3), and T4: 1 week (n=3). Additionally, control samples with magnetite-pine bark biosorbents (1g/L) were analysed after T1-SW: 3 h (n=1) and T2-SW: 24 h (n=1) of exposure to sterile water.

**References:**

Blanchard, G., Maunaye, M., Martin, G., 1984. Removal of heavy metals from waters by means of natural zeolites,. Water Res. 18, 1501–1507.

Lagergren, S., 1898. About the Theory of So-called Adsorption of Soluble Substances. K. Sven. Vetenskapsakad. Handingarl 24, 1–39.
